# The HIV care continuum and HIV-1 drug resistance among female sex workers: a key population in Guinea-Bissau

**DOI:** 10.1186/s12981-020-00290-3

**Published:** 2020-06-12

**Authors:** Jacob Lindman, Mamadu Aliu Djalo, Ansu Biai, Fredrik Månsson, Joakim Esbjörnsson, Marianne Jansson, Patrik Medstrand, Hans Norrgren, the SWEGUB CORE group, Babetida N’Buna, Antonio Biague, Ansu Biai, Cidia Camara, Zacarias Jose da Silva, Joakim Esbjörnsson, Marianne Jansson, Sara Karlson, Jacob Lindman, Patrik Medstrand, Fredrik Månsson, Hans Norrgren, Gülsen Özkaya Sahin, Sten Wilhelmson

**Affiliations:** 1 The Department of Clinical Sciences Lund, Lund University, Infektionskliniken Skånes Universitetssjukhus Lund, Hälsogatan 3, 221 85 Lund, Sweden; 2 Environmental Action in the Third World (ENDA), Bissau, Guinea-Bissau; 3 The National Public Health Laboratory, Bissau, Guinea-Bissau; 4 The Department of Translational Medicine, Lund University, Malmö, Sweden; 5 The Department of Laboratory Medicine, Lund University, Lund, Sweden

**Keywords:** Antiretroviral therapy, HIV care continuum, HIV seroprevalence, Sex workers, Africa, Drug resistance mutations

## Abstract

**Introduction:**

Female sex workers (FSW) are considered a key group for HIV transmissions in sub-Saharan Africa. The HIV Care Continuum and HIV drug resistance (HIVDR) among FSW has not been well studied in most countries in West Africa. In the current study we describe the HIV Care continuum and prevalence of HIVDR among FSW in Guinea-Bissau.

**Methods:**

A venue-based recruitment and peer-referral of FSW was used in seven cities in Guinea-Bissau from October 2014 to September 2017. We administered a questionnaire, performed discriminatory HIV-testing and collected blood specimens for CD4 count, viral load and HIVDR genotyping.

**Results:**

The survey included 440 FSW. The overall HIV-prevalence among FSW was 26.8%. Of the HIV-1 (HIV-1 single- or dually HIV-1/HIV-2) infected FSW (N = 104), 58.7% were previously diagnosed with HIV-1 at enrolment and 41.4% reported taking antiretroviral therapy (ART) compared to 28.6% of the HIV-2 single-infected FSW (N = 14). Among HIV-1 infected FSW on ART (N = 43), 55.8% were virally suppressed (< 1000 copies/ml) and of all HIV-1 infected FSW, 29.8% were virally suppressed. Among ART experienced FSW (N = 22), 50.0% had HIVDR. HIVDR was also found in 9.4% of treatment naïve FSW (N = 53).

**Conclusion:**

The majority of FSW who knew their HIV status received ART, however a large proportion of FSW were not aware of their HIV positive status. This translated into a great majority of the HIV-infected FSW not being virally suppressed. Amongst treatment naïve FSW nearly a tenth had HIVDR, suggesting that sexual transmission of HIVDR is occurring in this at-risk-population.

## Introduction

Female sex workers (FSW) play a key role for the transmission of HIV in sub-Saharan Africa [1]. The overall HIV prevalence among FSW in West and Central Africa was recently estimated to be 20.1% [2]. Antiretroviral therapy (ART) not only dramatically reduces HIV morbidity and mortality, but also eliminates infectiousness of treated HIV-infected individuals—referred to as treatment as prevention [3]. However, if individuals living with HIV should benefit from ART, they need to know that they are HIV infected, be linked to HIV care, and receive and adhere to effective ART. A systematic review of sub-Saharan African HIV treatment programs including over 58,000 patients diagnosed with HIV showed that linkage from HIV diagnosis to HIV care was poor [4]. In Guinea-Bissau, the national ART program was initiated in 2005 and according to UNAIDS about 49% of eligible women aged 15 and over were receiving ART in 2018 [5]. Unfortunately Guinea-Bissau has shown to be a country with generally poor ART adherence. In a report from an HIV clinic in the capital Bissau, between 2005 and 2012, more than half of the patients were lost to follow-up during the study period [6]. Side effects, food insecurity and travelling have been proposed as main barriers to ART non-adherence in Guinea-Bissau [7, 8]. As FSW are a marginalized group who are often stigmatized by health workers and also highly mobile, there is reason to believe that the disengagement from care could be even higher in this key population in Guinea-Bissau [9, 10]. A meta-analysis and systematic review of FSW in low-and-middle-income-countries estimated that current ART use among HIV-infected women was 39.2% (95% confidence interval (CI) 27.2–52.9%) [11]. However, the report also highlighted the lack of data. The HIV care continuum is an important framework to cross-sectionally describe the engagement in HIV care and treatment on a population level [12]. Previous studies among FSW in Malawi and Zimbabwe have found large numbers of drop-outs from the HIV care continuum [13, 14]. As more people are starting ART the risk of HIV drug resistance (HIVDR) mutations increases [15]. Various factors drive the emergence of HIVDR including poor adherence and logistical barriers [15, 16]. Even when adherence results in viral suppression HIVDR mutations may be acquired through multiple infections and sexual acquisition of transmitted drug resistance. The WHO´s 2017 drug resistance report showed that levels of pre-treatment drug resistance (PDR) were increasing in low and middle income countries between 2014 and 2016 [17]. This increase was mostly driven by increasing levels of non-nucleoside reverse transcriptase inhibitors (NNRTI) resistance which are currently used in many sub-Saharan countries as first-line ART regimens for HIV-1, including Guinea-Bissau [15]. A recent study of HIV-1 infected pregnant women in Guinea-Bissau reported a 10.4% prevalence of PDR to NNRTI [18]. There is currently little information on the HIV care continuum from diagnosis to viral suppression and emergence of HIVDR among FSW in West Africa. Without such estimates the unmet needs of this population with which to guide treatment programming is difficult to assess. Here, we report the HIV Care Continuum and HIVDR among FSW in seven cities in Guinea-Bissau.

## Methods

### Data collection

This cross-sectional study was designed and implemented through a collaboration between Lund University in Sweden, the National Public Health Laboratory (LNSP) in Guinea-Bissau, and Environmental Action in the third world (ENDA—a non-governmental organization in Guinea-Bissau).

Inclusion of participants took place between October 2014 and September 2017. The inclusion criteria were being biologically female, ≥ 16 years old, self-reported engagement in selling sex the last 12 months and giving oral and written informed consent to participate in the survey. Participants in the capital Bissau were included through venue-based sampling at nine different venues, which has documented success for identification of hidden populations [13, 19]. Participants in six cities outside Bissau were identified through peer-based chain-referral at one to four different venues depending on the size of the city (14 different venues in total) and were required to come to a mobile clinic at daytime. Peer-based chain-referral has previously been used to include FSW in studies in this region [19]. Participants were reimbursed for participating in the study ($2.67).

A team of one trained nurse, one social worker and one driver undertook the data collection. Interviewer-administered questionnaires was used to obtain information on demographics, HIV testing history, HIV care and treatment engagement, sex work, sexual behavior and condom use. Self-reported ART adherence was assessed by the visual analog scale (VAS) [20]. For the VAS, study participants were presented with line anchored at 0% and 100% and given examples of what 0%, 50% and 100% adherence represented. They were then asked to assess their ART adherence for the last 4 weeks [20]. Good ART adherence was defined as taking ≥ 90% of the daily medicine doses [20].

All women underwent venous blood sampling in a mobile clinic and were screened on site with an HIV-antibody rapid test, Determine HIV-1/2 (Abbott Diagnostic Division, Hoofdorp, Holland). The blood samples were stored in room temperature until transportation back to LNSP in Bissau the same day. At LNSP, a confirmatory discriminatory HIV test was performed using Immunocomb HIV1/2 BiSpot (Orgenics, Yavne, Israel) until September 2016, and thereafter Geenius 1/2 confirmatory assay (Biorad, Marnes-al-Coquette, France) as the production of Immunocomb ceased. Immunocomb and Geenius has been evaluated in parallel towards ability to discriminate between HIV-type [21]. CD4 measurement was obtained using the FACSPresto-Near Patient CD4 counter (Becton–Dickinson, NYSE:BDX, USA) [22]. Plasma was then separated from whole blood and until March 2016 all samples were transported on dry ice for HIV-1 viral load (VL) measurement and drug resistance genotyping at the Section of Clinical Virology at Lund University, Sweden (please see below for details). From March 2016, plasma HIV-1 viral load quantification was performed using GeneXpert at LNSP in Bissau [23]. The results of CD4 measurements and VL measurements were provided to participants at the next visit at the mobile clinic where the study nurse assisted with interpretation of the results. All HIV infected FSW were referred to an HIV treatment clinic of their choice together with their results for follow-up examination and treatment per national guidelines.

### HIV-1 plasma viral load determination and drug resistance genotyping

HIV-1 viral load was prior to March 2016 determined using an in-house TaqMan quantitative PCR (qPCR) with primers and probes as described [24]. qPCR was done using the ABI StepOnePlus System (Applied Biosystems) and the SuperScript III Platinum One-Step qRT-PCR Kit (Invitrogen) according to the manufacturer’s instructions. Polymerase chain reaction (PCR) conditions were as follows: 50 °C for 10 min and 95 °C for 2 min, followed by 40 cycles of 95 °C for 15 s, and 60 °C for 45 s. Stocks of HIV-1 strain IIIB which had been quantified by electron microscopy (Advanced Biotechnology Incorporated, Columbia MD), were used as standards. Known virus copy numbers were spiked into HIV-negative human plasma and extracted as previously described [24]. The qPCR assay had a limit of quantification of five RNA copies/qPCR reaction with a linear range between 10^6^ and 5 RNA copies/reaction.

HIV-1 drug resistance genotyping were determined as follows: HIV-1 RNA was extracted from blood plasma, and HIV-1 *pol* sequences were amplified by RT-PCR and nested PCR as described [25]. The final length of all the HIV-1 *pol* sequences following removal of regions corresponding to the primers, editing and alignment was 1035 bases, corresponding to nucleotide positions 2268–3302 of HXB2 (GenBank accession number K03455), covering positions 6-99 of the protease and positions 1-251 of the reverse transcriptase regions. The presence of drug resistance mutations (DRM) was assessed using the Stanford Genotypic Resistance Interpretation Algorithm [26]. DRM in self-reported treatment naïve individuals were examined according to the calibrated population resistance tool v6.0 beta (http://cpr.stanford.edu/cpr.cgi), based on the WHO surveillance transmitted drug resistance mutation list of 2009 [27, 28]. DRM in FSW on current ART and DRM in FSW reporting treatment discontinuation was assessed using The Stanford HIVdb program (https://hivdb.stanford.edu/hivdb/by-mutations/) [29].

### Statistics

The Chi square test was used for categorical variables and the two-tailed Mann–Whitney test U test for continuous variables. Trends in change of seroprevalence of HIV was analyzed by Cochran-Armitage test for linear trend adjusted for age. FSW were defined as virally suppressed with an HIV-1 VL < 1000 copies/ml [30, 31]. The continuum outcomes were characterized using two different approaches; using all HIV-1/dual-infected FSWs as the denominator or by using HIV-1/dual-infected FSW achieving the prior step in the continuum as the denominator. Logistic regression was used to assess predictors of outcomes in the HIV care cascade. Due to sample size, we were only able to investigate diagnosed HIV infection vs. undiagnosed HIV infection. Covariates were assessed in univariate and multivariate analysis. Covariates considered for inclusion in the multivariate model were age, region, education, condom use, number of clients last day of sex work, alcohol consumption and number of children. The final model was derived from an initial explanatory model with all variables included. Models were compared using likelihood ratio tests and Aikaki´s information criterion. In the final model the most important variables—age, region, education, condom use—were included. Data were managed in EPI INFO version 7.14 and analyzed by STATA 13 [32, 33].

## Results

**Fig. 1:**
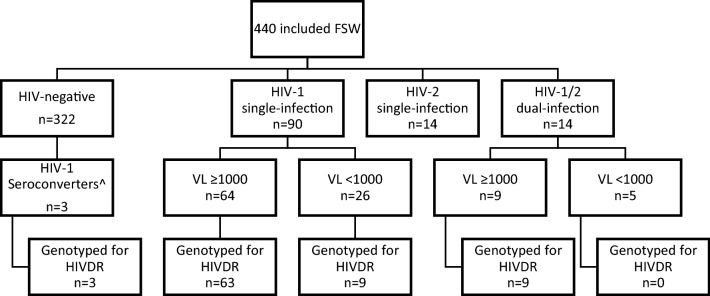
Flowchart of the study population. HIVDR, HIV drug resistance mutations; VL, Viral load. ^Three individuals seroconverting from HIV-seronegative to HIV-1 seropositive status during the study period

During the study period 847 women visited the mobile clinic. Among them, 490 women consented to participate in the study but 50 women did not engage in sex work according to our definition and were not included in the study. Thus a total of 440 women were included between October 2014 and September 2017, 63.2% of the women were included in the capital Bissau. Figure 1 shows the flowchart of the included study participants. The median age was 28 (Interquartile range (IQR) 22-35). The overall HIV-prevalence at inclusion was 26.8% (95% CI 22.7–31.2, n = 118). The total prevalence of HIV-1 single-infection, HIV-2 single-infection and HIV-1/2 dual infection was 20.5% (95% CI 16.8–24.5, n = 90), 3.2% (95% CI 1.8–5.3, n = 14) and 3.2% (95% CI 1.8–5.3, n = 14), respectively. The overall HIV-prevalence over the 3-year study period decreased over time from 32.6% (95% CI 25.6–40.1) between October 2014 and September 2015 (N = 172), 26.0% (95% CI 17.8––35.7) between October 2015 and September 2016 (N = 100), to 21.4% (95% CI 15.4-28.4) between October 2016 and September 2017 (N = 168) (p = 0.02 comparing the HIV prevalence over three time periods). After age-adjustments no statistically significant declining trend could be detected (p = 0.58 comparing the HIV prevalence over three time periods). Table 1 presents summary characteristics of HIV-infected participants stratified by HIV type and HIV-negative participants. HIV-1 single-infected FSW were younger (median age 30 years IQR 25–37) compared to both HIV-2 single-infected (38 years IQR 28–44, p = 0.03) and HIV-1/2 dual-infected (46.5 years IQR 35–49, p < 0.001) FSW but older than HIV-negative FSW (26 years IQR 22–32, p < 0.001). HIV-2 single-infected FSW had a higher median CD4 cell count (1039, IQR 727–1177) compared to both HIV-1 single-infected women (615 IQR 377–811 (p < 0.001)) and HIV-1/2 dual-infected women (525 IQR 296–741 (p = 0.001)), while there was no significant difference between HIV-1 single-infected women and HIV-1/2 dual-infected women (p = 0.38).

**Table 1:** Selected summary characteristics among HIV infected participants stratified by HIV type and HIV negative participants

	HIV-1 single-infected (n = 90)	HIV-1/2 dual-infected (n = 14)	HIV-2 single-infected (n = 14)	HIV negative (n = 322)	HIV-1 vs HIV negative p-value
Age	30 (25–37)	47 (35–49)	38 (28–44)	26 (22–32)	< .001^c^
CD4 cell count cells/mm^3^	615 (377–811)	525 (296–741)	1039 (727–1177)	NA	NA
HIV-1 VL copies/ml	4417 (631–30,193)	3138 (20–21,600)	NA	NA	NA
Region					.002^d^
Bissau	45 (50.0%)	11 (78.6%)	3 (21.4%)	103 (32.0%)	
Outside Bissau^a^	45 (50.0%)	3 (21.4%)	11 (78.6%)	219 (68.0%)	
Education (years)					.01^e^
None	33 (36.7%)	7 (50.0%)	8 (57.1%)	71 (22.1%)	
1–5	19 (21.1%)	54 (28.9%)	1 (7.1%)	52 (16.2%)	
6–9	22 (24.4%)	2 (14.3%)	2 (14.3%)	115 (35.7%)	
9–12	7 (7.8%)	0 (0.0%)	3 (21.4%)	71 (22.1%)	
Data missing	9 (10.0%)	1 (7.1%)	0 (0.0%)	13 (4.0%)	
Marital status					≤ .001^d^
Single	30 (33.3%)	3 (21.4%)	5 (35.7%)	207 (64.3%)	
Married	19 (21.1%)	2 (14.3%)	1(7.1%)	31 (9.6%)	
Divorce or widowed	37 (41.1%)	9 (64.3%)	8 (57.1%)	77 (23.9%)	
Data missing	4 (4.4%)	0 (0.0%)	0 (0.0%)	7 (2.2%)	
Number of clients per week					.08^d^
< 9	43 (47.8%)	7 (50.0%)	11 (78.6%)	196 (60.9%)	
≥ 10	33 (36.7%)	6 (42.9%)	2 (14.3%)	85 (26.4%)	
Data missing	14 (15.5%)	1 (7.1%)	1 (7.1%)	41 (12.7%)	
Condom use^b^					.22^d^
Inconsistent	43 (47.8%)	6 (42.9%)	9 (64.3%)	136 (42.2%)	
Consistent	42 (46.7%)	6 (42.9%)	4 (28.6%)	180 (55.9%)	
Data missing	5 (5.6%)	2 (14.2%)	1 (7.1%)	6 (1.9%)	

Data are n (%) or median (IQR)

*IQR* interquartile range, *NA* not applicable, VL viral load

^a^Canchungo, Bafata, Gabu, Bambadinca, Buba, Quebo

^b^Condom use last month; inconsistent including “Never” and “Sometimes”, consistent including “Always”

^c^Mann–Whitney test U test

^d^Chi Square test

^e^Kruskal–Wallis test

### HIV and the care continuum

**Fig. 2:**
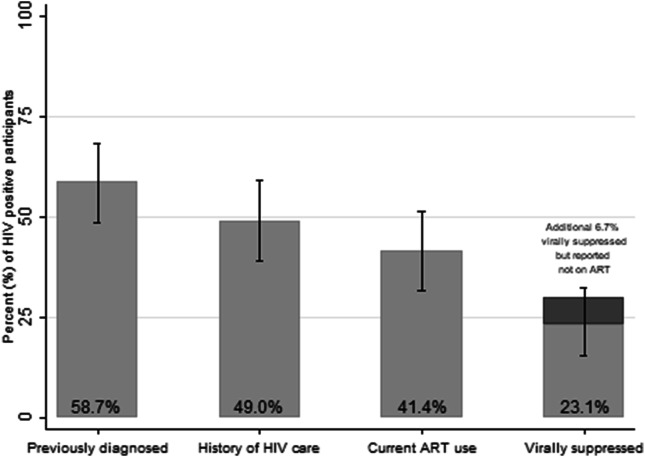
HIV care continuum among all HIV-1 infected FSW (n = 104). The y-axis and the figures in the bars indicate the proportion of all women testing HIV-1 positive. The proportion of women from each preceding step is described in the text

The HIV Care continuum among all HIV-1 infected FSW including HIV-1/2 dual-infected women is presented in Fig. 2 and in the following. Among the 104 HIV-1 infected FSW, 58.7*%* (95% CI 48.5–68.2, n = 61) knew their HIV status. The median CD4 cell count and VL was 559 cells/mm^3^ (IQR 322.5–751) and 2707 copies/ml (IQR 20–15849), respectively. The corresponding figures for HIV-1 infected FSW without a previous HIV diagnosis was 618 cells/mm^3^ (IQR 439–856) and 8110 copies/ml (IQR 2677–57800), respectively. Among the 14 HIV-2 single-infected FSW 64.3% (95% CI 35.1–87.2, n = 9) knew their HIV status. Parameters associated with undiagnosed HIV infection were determined by multivariable analysis. The analyses showed that younger FSW (< 30 years) were more likely to be previously undiagnosed (adjusted odds ratio (AOR) 2.7 (95% CI 1.2–6.5, p = 0.02), and there was some evidence that FSW residing outside Bissau were more likely to have an undiagnosed HIV infection (AOR 2.7 (95% CI 1.0–6.8, p = 0.05)). More importantly, an unknown HIV diagnosis was associated with inconsistent condom use with clients (AOR 3.0 (95% CI 1.04–8.9, p = 0.04) (Table 2).

**Table 2:** Association between sociodemographic factors and transmission risk factors with a previously undiagnosed HIV infection

	n	Undiagnosed HIV infection	OR	95% CI	p	AOR^a^	95% CI	p
HIV-type								
HIV-1	90	40 (44.4%)	Ref					
HIV-2	14	5 (35.7%)	0.7	0.2–2.2	0.54			
HIV-1/2 dual infected	14	3(21.4%)	0.3	0.1–1.3	0.12			
Age								
≥ 30	73	23 31.5%)	Ref			Ref		
< 30	45	25 (55.6%)	2.7	1.3–5.9	0.01	2.7	1.2–6.5	0.02
Nationality								
Guinea-Bissau	109	44 (40.4%)	Ref					
Other^b^	9	4 (44.4%)	1.2	0.3–4.7	0.81			
Region								
Bissau	59	18 (30.5%)	Ref			Ref		
Outside Bissau^c^	59	30 (50.9%)	2.4	1.1–5.1	0.03	2.4	1.0-5.8	0.05
Marital status								
Single	38	15 (39.5%)	Ref					
Married	22	8 (36.4%)	0.9	0.3–2.6	0.81			
Divorced/widowed	54	22 (40.7%)	1.1	0.5–2.5	0.90			
Data missing	4	3 (75.0%)						
Children								
≥ 1 child	98	36 (36.7%)	Ref					
None	16	10 (62.5%)	2.9	1.0–8.6	0.06			
Data missing	4	2 (50.0%)						
Education (years**)**								
None	48	18 (37.5%)	Ref			Ref		
1–5	24	9 (37.5%)	1	0.4–2.8	1.0	Ref		
6–9	26	9 (34.6%)	0.9	0.3–2.4	0.81	Ref		
10–12	10	7 (70.0%)	3.9	0.9–16.7	0.07	4.2	0.9–19.9	0.08
Data missing	10	5 (50.0%)						
Another source of income^d^								
No	61	22 (36.1%)	Ref					
Yes	53	25 (47.2%)	1.6	0.7–3.4	0.23			
Data missing	4	1 (25.0%)						
Number of clients^e^								
≥ 2	40	14 35.0%)	Ref					
0–2	62	30 (48.4%)	1.7	0.8–3.9	0.18			
Data missing	16	4 (25.0%)						
Condom use with client^f^								
Consistent	85	31 (36.5%)	Ref			Ref		
Inconsistent	23	37 (60.9%)	2.7	1.1–7.0	0.04	3.0	1.04–8.4	0.04
Data missing	10	3 (30.0%)						
Alcohol comsumption^g^								
Never	74	35 (47.3%)	Ref					
Yes	40	12 (30.0%)	0.5	0.2–1.1	0.08			
Data missing	4	1(25.0%)						

*AOR* adjusted odds ratio, *CI* confidence interval, *OR* odds ratio, *p* p-value, *Ref* reference category

^a^In the final multivariate model the following variables were included; age, region, education (0–9 years vs 10–12 years), and condom use

^b^Including Gambia and Senegal

^c^Including Canchungo, Bafata, Gabu, Bambadinca, Buba, Quebo

^d^Besides sex work

^e^Number of clients last day of sex work

^f^Do you accept condomless sex with clients?; inconsistent including “Yes”, Consistent including “No”

^g^Alcohol consumption last month; Yes including “Every day”, “Once a week”, “less than once a week”, No including “Never”

A history of HIV care was reported in 49.0% (95% CI 39.1–59.0, n = 51) of all HIV-1 infected FSW (Fig. 1), and in 83.6% (95% CI 71.9–91.8, n = 51) of the FSW previously diagnosed with an HIV-1 infection. Among the HIV-2 single-infected FSW 28.6% (95% CI 8.4–58.1, n = 4) reported a history of HIV care, of whom all reported current ART use. Of all HIV-1 infected FSW, 41.4% (95% CI 31.7–51.4, n = 43) reported taking ART compared to 84.3% (95% CI 71.4–92.9, n = 43) of HIV-1 infected FSW with a history of HIV care and 70.5% (95% CI 57.4–81.4, n = 43) of HIV-1 infected FSW with a previous diagnosis. Among the HIV-1 infected FSW reporting current ART use, 55.8% (95% CI 39.9–70.9, n = 24) were virally suppressed, which was 23.1% (95% CI 15.4–32.4, n = 24) of all women testing HIV-1 positive. An additional 6.7% (95% CI 2.7–13.4, n = 7) of HIV-1 infected women were virally suppressed despite not reporting ART use, which means that a total of 29.8% of HIV-1 infected women (95% CI 21.2–39.6, n = 31) were virally suppressed. Self-reported adherence ≥ 90% measured by VAS, among 27 of the HIV-1 infected FSW reporting current ART use, was associated with viral suppression after age adjustments (odds ratio 20.0, 95% CI 2.5–161.7, p = 0.01).

### HIV-1 resistance mutations

All resistance mutations identified in HIV-1 infected FSW with a viral load ≥ 1000 copies/ml are summarized in Table 3. Among HIV-1 infected FSW with a viral load < 1000 copies/ml no HIVDR were detected in the nine samples that were analyzed for HIVDR. At least one HIVDR was detected in 9.4% (95% CI 3.1–20.7, n = 5) of the 53 treatment naïve FSW, as compared with 50.0% (95% CI 28.2–71.8, n = 11) of 22 ART experienced FSW. Among the treatment naïve FSW, 6.0% (95% CI 1.2–15.7, n = 3) had NNRTI mutations, and 4.0% (95% CI 0.5–13.0, n = 2) had nucleoside reverse transcriptase inhibitor (NRTI) mutations. Among the 22 ART experienced FSW, NNRTI only resistance mutations were detected in 18.2% (95% CI 5.2–40.3, n = 4), NRTI only resistance mutations in 9.1% (95% CI 1.1–29.2, n = 2) and NNRTI + NRTI mutations were detected in 18.2% (95% CI 5.2–40.3, n = 4). One individual had a protease inhibitor accessory mutation. There was no difference in time on ART between FSW with or without HIVDR (3.6 IQR 1.7-4.4 and 3.2 years IQR 1.8–6.1, respectively, p = 0.59).

**Table 3:** Summary characteristics by treatment status among HIV-1 infected participants with viral load ≥ 1000 copies/ml

	Treatment naive^a^ (n = 53)	ART expererienced^b^ (n = 22)	Treatment Naive vs ART experienced p-value
Age	30 (24–35)	42 (30–46)	.002^c^
HIV-1/2 dual-infected	4 (7.6%)	5 (22.7%)	0.07^d^
CD4 cells/mm^3^	584 (373–787)	432 (184–726)	.17^c^
HIV-1 VL copies/ml	25,461 (5649–59,182)	7130 (2707–21,771)	.01^c^
Previous diagnosis	14 (27.5%)	22 (100.0%)	NA
Non-mutated virus	48 (90.6%)	11 (50.0%)	
Detectable resistance	5 (9.4%)	11 (50.0%)	< .001^d^
NNRT^I^ only	3 (5.7%)	4 (18.2%)	NA
NRTI only	2 (3.8%)	2 (9.1%)	NA
NRTI + NNRTI	0 (0.0%)	4 (18.1%)	NA
PI minor	0 (0.0%)	1 (4.6%)	NA

Data are n (%) or median (IQR)

*ART* antiretroviral therapy, *NNRTI* non-nucleoside reverse transcriptase inhibitors, *NRTI* nucleoside reverse transcriptase inhibitor, *NA* not applicable, *PI* protease inhibitors, *VL* viral load

^a^Including 3 seroincident individuals

^b^Including 3 individuals with previous ART use

^c^Mann–Whitney test U test

^d^Chi Square test

Table 4 shows all resistance mutations by subset in the 16 FSW with a HIVDR. Overall, the most common NNRTI resistance mutation was K103N which was found in 43.8% (95% CI 19.7–70.1, n = 7) of FSW with a HIVDR mutation. The most common NRTI mutation was M184V which was found in 37.5% (95% CI 15.2–64.6, n = 6) of FSW with a HIVDR mutation. By splitting the study period in two 18.5-month time strata; September 2014 to 15th March 2016 and 16^th^ March 2016 to September 2017, we investigated if any temporal trends in prevalence of PDR among treatment naïve FSW could be seen. The prevalence of PDR were 4.2% (95% CI 0.1–21.1, n = 1) among 24 treatment naïve FSW included in the first time-period and 13.8% (95% CI 3.9–31.6, n = 4) among 29 treatment naïve FSW included in the last time-period (p = 0.26 comparing the prevalence in the two time periods).

**Table 4:** HIVDR mutations by treatment status among 16 FSW with detectable HIVDR in Guinea-Bissau

Drug class	HIVDR Mutation (n = 16)	Treatment Naive (n = 5)	ART experienced^a^ (n = 11)	Total (n = 16)
NNRTI	K103N/S	3 (60.0%)	4 (36.4%)	*7 (43.8%)*^*b*^
	A98G	0 (0.0%)	2 (18.2%)	2 (12.5%)
	E1238A	0 (0.0%).	1 (9.1%)	1 (6.3%)
	K238N	0 (0.0%)	1 (9.1%)	1 (6.35)
	K238T	0 (0.0%)	1 (9.1%)	1 (6.3%)
	P225H	0 (0.0%)	1 (9.1%)	1 (6.3%)
	V106A	0 (0.0%)	1 (9.1%)	1 (6.3%)
	V179E	0 (0.0%)	1 (9.1%)	1 (6.3%)
	Y188H	0 (0.0%)	1 (9.1%)	1 (6.3%)
	Y188L	1 (20.0%)	0 (0.0%)	1 (6.3%)
NRTI	M184V	0 (0.0%)	6 (54.5%)	*6 (37.5%)*^*b*^
	M41L	1 (20.0%)	1 (9.1%)	2 (12.5%)
	T215Y	0 (0.0%)	2 (18.2%)	2 (12.5%)
	D67N	1 (20.0%)	0 (0.0%)	1 (6.3%)
	E44D	0 (0.0%)	1 (10.0%)	1 (6.3%)
	K219E	1 (20.0%)	0 (0.0%)	1 (6.3%)
	T215FY	0 (0.0%)	1 (9.1%)	1 (6.3%)
	T215I	1 (20.0%)	0 (0.0%)	1 (6.3%)
PI	L89V	0 (0.0%)	1 (9.1%)	*1 (6.3%)*^*b*^

Data are n (%)

*ART* antiretroviral therapy, *HIVDR* HIV-1 drug resistance, *NNRTI* non-nucleoside reverse transcriptase inhibitors, *NRTI* nucleoside reverse transcriptase inhibitor, *PI* protease inhibitors

^a^Including 10 study participants with current ART use and 1 study participant with previous ART use

^b^The most common HIVDR in each drug class highlighted in italics

## Discussion

In this study, we investigated the HIV care continuum and HIV-1 drug resistance among 440 FSW across six sites in Guinea-Bissau. The overall HIV prevalence was 26.8%, with nearly four times higher total HIV-1 prevalence (23.6%) than total HIV-2 prevalence (6.4%) when including HIV-1/2 dual-infected FSW in each group. The comparably low HIV-2 prevalence is in concordance with the decreasing HIV-2 prevalence that has been observed in an occupational cohort of police officers, among pregnant women and the general population. [34–36] The prevalence of HIV-1 was more than four times higher while the HIV-2 prevalence was nearly two times higher among the FSW observed in our study compared to adult women in the most recent community-based study from Bissau, were the HIV-1 and HIV-2 prevalence was 5.2% and 3.4% respectively [35]. The CD4+ T cell levels were generally high, also among FSW not previously diagnosed, probably due to a relatively short duration of infection among most participants.

Our finding that more than one-third of all HIV-1 infected FSW and HIV-2 infected FSW were not aware of their HIV infection is considerably higher than reported in studies from Malawi, South Africa and Zimbabwe [13, 14, 37]. This is a concern because individuals that are unaware of their HIV status will not be linked to HIV care. Another concern is the increased risk of onward HIV transmission since FSW unaware of their HIV infection were more likely to have inconsistent condom use with clients. Currently, WHO recommends HIV testing every 12 months for FSW [38]. It is therefore plausible that frequent testing strategies for FSW would be beneficial in Guinea-Bissau, particularly among younger FSW and FSW residing outside the capital Bissau.

A meta-analysis and systematic review among FSW in low-and-middle-income-countries found an estimated ART coverage of 39.3% (27.2–52.9%) [11]. The ART coverage among all HIV-1 infected FSW in our study was 41.4%, which is comparable to this meta-analysis as well as other studies of FSW from Zimbabwe (43.4%) and South Africa (43.9%), but lower than in Malawi (52.2%) [13, 14, 37]. The corresponding ART coverage for HIV-2 single-infected women in our study was 28.6%. We recently showed that HIV-2 is more pathogenic than previously thought and that the majority of HIV-2 infected individuals will progress to HIV-related disease and death without treatment, further emphasizing the importance of high ART coverage among HIV-2 infected individuals [39].

The status of the UNAIDS goals among the general population of women of the same age in Guinea-Bissau have not been detailed. However, the ART coverage among FSW in our study did not reach the 90-90-90 goals set by UNAIDS but among FSW on ART 55.8% were virally suppressed, indicating that the majority of FSW are likely to be adherent to their ART regime [40]. Moreover, more than half of the FSW on treatment that failed to suppress their virus had at least one HIVDR mutation. It is likely that many of these mutations represent acquired resistance mutations due to non-adherence, but given our findings regarding PDR, it is possible that some of these mutations represents transmitted drug resistance. In nearly half of the FSW on treatment with viremia, non-mutated virus was identified which suggest treatment interruptions as the cause of their virological failure. Other explanations could be problems with drug dosing or absorption, as previously described by Lessells et al. [41].

Total HIVDR in our study (as percentage of all HIV-1 infected participants) was in line with a recent study from South Africa (15.4% vs 16.7%) [37]. Similarly to our study, the report from South Africa included both FSW on treatment, FSW reporting treatment discontinuation, and treatment naïve FSW. The most common resistance mutation in the ART naïve FSW was the *K103N* and *K103S* mutations, which is similar to a recent study of pregnant women in Guinea Bissau [25]. The prevalence of PDR was high, especially in the second part of the study were the prevalence reached 13.8% although with a large uncertainty of the estimate. This is worrisome and further studies are necessary to investigate if PDR is increasing in Guinea-Bissau. Moreover, the most frequent mutations found in our study has also been reported to be common among patients failing first line ART in the country [42].

Female sex workers are a highly mobile and vulnerable population in Guinea-Bissau. We therefore used a mixed venue-based and chain-referral convenience sampling. Hence, it is possible that our study population is not representative of all FSW in Guinea-Bissau. In addition, we could not adjust for clustering by recruitment chain or venue, other than city, because data on which participants were recruited by whom or from where were not recorded. It is possible that Respondent Driven Sampling could have addressed these difficulties, but due to budget and resource limitations, we decided to use the more cost-effective mixed venue-based and chain-referral convenience sampling. Information on HIV care continuum outcomes mainly relied on self-report, and thus subject to social desirability and misclassification. Of the HIV positive women reporting not being on ART, 6.7% had a VL < 1000 copies/ml. Discrepancies between viral suppression and self-reporting of not being on ART has been shown also in other studies of FSW [13, 14]. The most likely explanation is misclassification, but it could also be possible that a small proportion of these women were elite controllers [21, 43]. Regarding HIVDR data, the sample was small and therefore limited the generalizability. Moreover, it cannot be excluded that women reporting not being on ART could have previously received PMTCT, even though fieldworkers probed for this.

## Conclusion

This is the first study to characterize the HIV care continuum among FSW in Guinea-Bissau. Our study suggests that FSW are highly burdened to HIV compared to adult women in Guinea-Bissau, and of all HIV-1 infected women only a third were virally suppressed. We also found high levels of HIVDR mutations among both treatment naïve FSW and FSW on ART. Future studies are needed to assess HIV prevalence and HIVDR mutations among FSW and other most-at-risk-populations (MARPs) in the region. Such studies would be pivotal to inform future targeted interv against MARPs in resource-limited settings like Guinea-Bissau.

## Data Availability

The dataset used for the current study is available from the corresponding author on reasonable request

## Abbreviations

FSW: Female sex workers
HIVDR: HIV drug resistance
ART: Antiretroviral therapy
PDR: Pre-treatment drug resistance
NNRTI: Non-nucleoside reverse transcriptase inhibitors
LNSP: The National Public Health Laboratory
ENDA: Environmental action in the third world
VAS: Visual analog scale
VL: Viral load
qPCR: Quantitative PCR
PCR: Polymerase chain reaction
DRM: Drug resistance mutations
AOR: Adjusted odds ratio
NRTI: Nucleoside reverse transcriptase inhibitor
MARPs: Most-at-risk-populations

## References

[1] Baral S, Beyrer C, Muessig K, Poteat T, Wirtz AL, Decker MR (2012). Burden of HIV among female sex workers in low-income and middle-income countries: a systematic review and meta-analysis. Lancet Infect Dis.

[2] Shannon K, Crago A-L, Baral SD, Bekker L-G, Kerrigan D, Decker MR (2018). The global response and unmet actions for HIV and sex workers. Lancet.

[3] Lundgren JD, Babiker AG, Gordin F, Emery S, Grund B, Group ISS (2015). Initiation of antiretroviral therapy in early asymptomatic HIV infection. N Engl J Med.

[4] Mugglin C, Estill J, Wandeler G, Bender N, Egger M, Gsponer T (2012). Loss to programme between HIV diagnosis and initiation of antiretroviral therapy in sub-Saharan Africa: systematic review and meta-analysis. Trop Med Int Health..

[5] UNAIDS. Country factsheets Guinea-Bissau. 2018.

[6] Honge BL, Jespersen S, Nordentoft PB, Medina C, da Silva D, da Silva ZJ (2013). Loss to follow-up occurs at all stages in the diagnostic and follow-up period among HIV-infected patients in Guinea-Bissau: a 7-year retrospective cohort study. BMJ Open.

[7] Dyrehave C, Rasmussen DN, Honge BL, Jespersen S, Correia FG, Medina C (2016). Nonadherence is associated with lack of HIV-related knowledge: a cross-sectional study among HIV-infected individuals in guinea-bissau. J Int Assoc Provid AIDS Care..

[8] Nordentoft PB, Engell-Sorensen T, Jespersen S, Correia FG, Medina C, da Silva Te D (2017). Assessing factors for loss to follow-up of HIV infected patients in Guinea-Bissau. Infection.

[9] Scambler G, Paoli F (2008). Health work, female sex workers and HIV/AIDS: global and local dimensions of stigma and deviance as barriers to effective interventions. Soc Sci Med.

[10] Ramesh S, Ganju D, Mahapatra B, Mishra RM, Saggurti N (2012). Relationship between mobility, violence and HIV/STI among female sex workers in Andhra Pradesh, India. BMC Public Health..

[11] Mountain E, Mishra S, Vickerman P, Pickles M, Gilks C, Boily MC (2014). Antiretroviral therapy uptake, attrition, adherence and outcomes among HIV-infected female sex workers: a systematic review and meta-analysis. PLoS ONE.

[12] Gardner EM, McLees MP, Steiner JF, Del Rio C, Burman WJ (2011). The spectrum of engagement in HIV care and its relevance to test-and-treat strategies for prevention of HIV infection. Clin Infect Dis.

[13] Lancaster KE, Powers KA, Lungu T, Mmodzi P, Hosseinipour MC, Chadwick K (2016). The HIV care continuum among female sex workers: a key population in Lilongwe, Malawi. PLoS One..

[14] Cowan FM, Davey CB, Fearon E, Mushati P, Dirawo J, Cambiano V (2017). The HIV care cascade among female sex workers in Zimbabwe: results of a population-based survey from the sisters antiretroviral therapy programme for prevention of HIV, an integrated response (SAPPH-IRe) trial. JAIDS.

[15] WHO (2017). HIV drug resistance report.

[16] Stadeli KM, Richman DD (2013). Rates of emergence of HIV drug resistance in resource-limited settings: a systematic review. Antivir Ther..

[17] HIV drug resistance report 2017. Geneva: World Health Organization; 2017. Licence: CC BY-NC-SA 3.0 IGO.

[18] Wilhelmson S, Mansson F, Lopatko Lindman J, Biai A, Esbjornsson J, Norrgren H (2018). Prevalence of HIV-1 pretreatment drug resistance among treatment naive pregnant women in Bissau, Guinea Bissau. PLoS One..

[19] Peitzmeier S, Mason K, Ceesay N, Diouf D, Drame F, Loum J (2014). A cross-sectional evaluation of the prevalence and associations of HIV among female sex workers in the Gambia. Int J STD AIDS.

[20] Amico KR, Fisher WA, Cornman DH, Shuper PA, Redding CG, Konkle-Parker DJ (2006). Visual analog scale of ART adherence: association with 3-day self-report and adherence barriers. JAIDS.

[21] Lindman J, Hønge BL, Kjerulff B, Medina C, da Silva ZJ, Erikstrup C (2019). Performance of Bio-Rad HIV-1/2 confirmatory assay in HIV-1, HIV-2 and HIV-1/2 dually reactive patients—comparison with INNO-LIA and immunocomb discriminatory assays. J Virol Methods..

[22] Angira F, Akoth B, Omolo P, Opollo V, Bornheimer S, Judge K (2016). Clinical evaluation of the BD FACSPresto near-patient CD4 counter in Kenya. PLoS One..

[23] Kulkarni S, Jadhav S, Khopkar P, Sane S, Londhe R, Chimanpure V (2017). GeneXpert HIV-1 quant assay, a new tool for scale up of viral load monitoring in the success of ART programme in India. BMC Infect Dis.

[24] Özkaya Şahin G, Månsson F, Palm AA, Vincic E, da Silva Z, Medstrand P (2013). Frequent intratype neutralization by plasma immunoglobulin a identified in HIV type 2 infection. AIDS Res Hum Retrovir.

[25] Wilhelmson S, Månsson F, Lopatko Lindman J, Biai B, Esbjörnsson J, Norrgren H (2018). Prevalence of HIV-1 pretreatment drug resistance among treatment naïve pregnant women in Bissau. PloS One..

[26] Stanford University HIV Drug resistance Database (2018). HIVbd program genotypic resistance interpretation algorithm.

[27] Bennett DE, Camacho RJ, Otelea D, Kuritzkes DR, Fleury H, Kiuchi M (2009). Drug resistance mutations for surveillance of transmitted HIV-1 drug-resistance: 2009 update. PLoS One..

[28] Gifford RJ, Liu TF, Rhee SY, Kiuchi M, Hue S, Pillay D (2009). The calibrated population resistance tool: standardized genotypic estimation of transmitted HIV-1 drug resistance. Bioinformatics..

[29] Shafer RW (2006). Rationale and uses of a public HIV drug-resistance database. J Infect Dis..

[30] WHO (2016). Consolidated guidelines on the use of antiretroviral drugs for treating and preventing HIV infection: recommendations for a public health approach.

[31] WHO (2015). Consolidated strategic information guidelines for HIV in the health sector.

[32] Dean A, Arner T, Sunki G, Friedman R, Lantinga M, Sangam S (2011). Epi Info™, a database and statistics program for public health professionals.

[33] Statacorp LP (2013). Stata statistical software: release 13.

[34] Mansson F, Biague A, da Silva ZJ, Dias F, Nilsson LA, Andersson S (2009). Prevalence and incidence of HIV-1 and HIV-2 before, during and after a civil war in an occupational cohort in Guinea-Bissau. West Africa. AIDS..

[35] Olesen JS, Jespersen S, Da Silva ZJ, Rodrigues A, Erikstrup C, Aaby P (2018). HIV-2 continues to decrease, while HIV-1 is stabilizing in Guinea-Bissau. AIDS..

[36] Mansson F, Alves A, Silva ZJ, Dias F, Andersson S, Biberfeld G (2007). Trends of HIV-1 and HIV-2 prevalence among pregnant women in Guinea-Bissau, West Africa: possible effect of the civil war 1998 1999. Sex Transm Infect..

[37] Coetzee J, Hunt G, Jaffer M, Otwombe K, Scott L, Bongwe A (2017). HIV-1 viraemia and drug resistance amongst female sex workers in Soweto, South Africa: a cross sectional study. PLoS One..

[38] WHO (2019). Consolidated guidelines on HIV testing services for a changing epidemic.

[39] Esbjörnsson J, Månsson F, Kvist A, da Silva ZJ, Andersson S, Fenyö EM (2019). Long-term follow-up of HIV-2-related AIDS and mortality in Guinea-Bissau: a prospective open cohort study. Lancet HIV..

[40] UNAIDS (2014). 90-90-90: An Ambitious Treatment Target To Help End the AIDS eoidemic.

[41] Lessells RJ, Avalos A, de Oliveira T (2013). Implementing HIV-1 genotypic resistance testing in antiretroviral therapy programs in Africa: needs, opportunities, and challenges. AIDS Rev..

[42] Rhee SY, Blanco JL, Jordan MR, Taylor J, Lemey P, Varghese V (2015). Geographic and temporal trends in the molecular epidemiology and genetic mechanisms of transmitted HIV-1 drug resistance: an individual-patient- and sequence-level meta-analysis. PLoS Med..

[43] Deeks SG, Walker BD (2007). Human immunodeficiency virus controllers: mechanisms of durable virus control in the absence of antiretroviral therapy. Immunity..

